# Peer review of "ORP5 and ORP8: Sterol Sensors and Phospholipid Transfer Proteins at Membrane Contact Sites?"

_biomolecules, 2020, doi:10.3390/biom10060928_

Round 1
Reviewer 1 Report
In their review paper, Nunes-Hasler and co-workers describe the current knowledge on ORP5/ORP8 , showing that is remains difficult to perceive what their exact cellular function(s) are. They clearly make the point that one of the question hinges on whether these proteins transfer sterol in addition to PS and PI4P. This review is informative and well documented, well-written and I do acknowledge the efforts made by the authors who tried to offer a comprehensive digest from all the data, which can be very contradictory, obtained so far on ORP5/ORP8 proteins.
I think that a few points of the review paper can be improved to be suitable for publication and that the authors should be more precise on biochemical/structural aspects and even be quite more critical on some studies. I agree that old data are enigmatic and must be explained but I do believe that ORP5/ORP8 do not transfer sterol. Closest homologues Osh6/7 neither extract nor transfer sterol in vitro (Prinz and co-workers, JCB, 2009, Moser Von Filseck, Science, 2015. Du and co-workers reported that ORP5 transfers sterol in vitro but the transfer rate is 30 molecule of sterol/ hour/ molecule, which is absolutely slow. This is comparable to the activity of Osh4 but the activity of Osh4 in that case is measured in the absence of its counterligand and is likely 10-times slower than what can be expected based on other works. So what is measured is almost noise. Also as mentioned by the authors, Maeda and De Camilli’s teams did not detect sterol in the pocket of Osh6/7 and ORP8 when these proteins are extracted from a cellular context. Recent data showing that Osh6/7 have more affinity for sterol than Osh4 are quite curious.
Overall comment: the authors must clearly define throughout the text what they mean by "lipid binding". The ORD surface can bind to lipids present in a membrane to associate (not specifically) with this membrane or can specifically extract and transfer a lipid (PS or PI4P) - This distinction is not often made in the manuscript or in the field in general. Yet this is quite fundamental. Second, some structural data completely contradict some old or more recent claims and this should be said.
About the conclusion section, authors might consider two additional points.
- PI4P is a ligand for ORP5/ORP8 but also for OSBP and ORP1. Thus a lack of ORP5/8 might somehow impact PI4P level in the endosomal/lysosomal compartment. In turn this might change the activity of OSBP and/or ORP1 and sterol level in the limiting membrane of endosomes/lysosomes – recent papers showed intriguing links between OSBP and retromer or mTORC1 at endosome/lysosome levels . Authors should discuss this point.
- There is a special link between PS and sterol. These lipids tend to coalesce together (Fairn’s papers). Authors should shortly discuss whether a misdelivery of PS could in turn change how sterol is distributed in some compartments, again to interpret old data.
Last, authors should more precisely say what could be the sensor role of ORP5/8 – Because there is no mechanistic data on that, this concept remains quite obscure.
P2 line 61-66
These sentences are a little fuzzy, notably on how sterol transfer by Osh4/OSBP relies on PIPs . Prinz and co-workers showed that Osh4 transfers more rapidly sterol in the presence of PIP2 simply because PIP2 provides negative charge to membrane, not because PIP2 is a counterligand. Authors should provide the entire story : (i) the identification of Osh4 as a protein able to extract sterol or PI4P and not simply as a protein that binds to a PIP-rich membrane (de Saint-Jean et al, 2011) and (ii) the work on OSBP. Of note the study on OSBP did not show that OSBP transfers sterol against its gradient by counterexchange with PI4P. This has been only formally demonstrated using Osh4 by Moser von Filseck et al in Nature Com, 2015.
P2 line 73-74 : The authors must precise whether they have aligned sequences or compare structures to get this conclusion.
P2 line 80. I think that this amphipathic helix cannot help ORD5 to target LDs simply because this AH is part of the ORP ORD5 core structure and is deeply embedded inside this structure according to any robust molecular model based on homology with Osh6/Osh7. I think that the JCB paper on LD and ORP5 is convincing but there is a big problem in term of structural analysis. I will be very cautious on this.
Figure 1 : panel C. There is definitively no coiled-coil region in Osh6/Osh7 according to the PDB structures of Osh6. I think the figure must be corrected and that the corresponding paper in EMBO J must be re-discussed according to the structural data that have been obtained later by Maeda and co-workers.
Figure1 : panel A – I would say ORD signature motif
P5 line 186 : typo
P6 –line 212 : this paper must be re-analyzed according to the structural data on Osh6 (see previous comment)
P6 – line 236 “dihydroergosterol “ à “dehydroergosterol”
P6 – line 249 : typo ‘the whether”
P6 – line 255 : typo ‘in-vitro’
P6 – line255-256 and following sentences in page 7 : To me, assays showing the binding of Osh6/Osh7 to phospholipid spotted on nitrocellulose or even included in liposomes are not indicative of an ability to regulate these phospholipids. Only the work of Maeda and co-workers showed first an ability of Osh6/7 to regulate PS. Also, back to my overall comment, I would say “ phospholipid encapsulation by ORP5” , “the role of Osh6 in capturing..” instead of “binding”
P7 – line 267 – the two back-to-back papers were published in Science
P7 –line 300-304. It must be said that, comparatively to the DeCamilli’s paper, that this paper reported clear measurement of PS transfer from the ER to the PM and the dependence of this process on a PI4P gradient.
P7 – line 307 – I don’t understand the use of the word “co-exchanger”
P8 – line 318 : typo “asses”
P8 – line 345-360 – No doubt that the T Balla’s work is beautiful and decisive but please note that the De Camilli’s group had already replaced the PH domain of ORP5/8 by a FKBP module in its Science paper, showing that the recruitment of these proteins upon adding rapamycin elicits a change in PM PS and PI4P level, thereby validating the PS/PI4P exchange model. Authors should correct this paragraph.
P9 –line 367 – typo “PIP5kIb”
P9 – line 380 – I think that ORP8 does not replace ORP5 but is recruited at the MCSs to work in addition to ORP5 when the level of PIP2 rises at the PM.
Figure 2 : panel A : I would not draw a PH domain like that , binding two PIPs at the same time – I think this is wrong from a structural point of view and misleading.
Please note that the names used to designate PIPs are not the same throughout the manuscript.
Author Response
Please find below our point-by-point response in italics. The page and line numbers correspond to the file with "tracked changes" on with the option "Show all revisions in line"
In their review paper, Nunes-Hasler and co-workers describe the current knowledge on ORP5/ORP8 , showing that is remains difficult to perceive what their exact cellular function(s) are. They clearly make the point that one of the question hinges on whether these proteins transfer sterol in addition to PS and PI4P. This review is informative and well documented, well-written and I do acknowledge the efforts made by the authors who tried to offer a comprehensive digest from all the data, which can be very contradictory, obtained so far on ORP5/ORP8 proteins.
We thank the reviewer for these positive comments and for such a careful reading of our manuscript. We are happy to see that our review has stimulated the reviewer to write such a detailed discussion, and hope they will find the new manuscript improved.
I think that a few points of the review paper can be improved to be suitable for publication and that the authors should be more precise on biochemical/structural aspects and even be quite more critical on some studies. I agree that old data are enigmatic and must be explained but I do believe that ORP5/ORP8 do not transfer sterol. Closest homologues Osh6/7 neither extract nor transfer sterol in vitro (Prinz and co-workers, JCB, 2009, Moser Von Filseck, Science, 2015. Du and co-workers reported that ORP5 transfers sterol in vitro but the transfer rate is 30 molecule of sterol/ hour/ molecule, which is absolutely slow. This is comparable to the activity of Osh4 but the activity of Osh4 in that case is measured in the absence of its counterligand and is likely 10-times slower than what can be expected based on other works. So what is measured is almost noise. Also as mentioned by the authors, Maeda and De Camilli’s teams did not detect sterol in the pocket of Osh6/7 and ORP8 when these proteins are extracted from a cellular context. Recent data showing that Osh6/7 have more affinity for sterol than Osh4 are quite curious.
We respect the reviewer’s opinion that ORP5/8 may simply not transfer sterol, as this may very well be true, and agree with the reviewer that the in vitro data presented by Du and colleagues are by no means conclusive. We have now put more emphasis on these doubts in the revised manuscript as suggested. Pg17 lines 557-561, 581-588
However, we respectfully disagree that a measurement that is 6-fold higher compared to negative control is almost noise, this is a bit unfair. Even if other LTPs such as Osh4 have a much higher transfer rate with an optimal mixture of lipid ligands, by the same rationale could it not be argued then that the slow transfer rate observed for ORP5 could also be due to the fact that the reaction conditions were not optimal? Perhaps ORP5 requires a protein cofactor/interactor (for example NPC1) or post-translational modification that is not present in the liposome preparation. The argument that the Gavin and De Camilli teams did not detect sterol in the pocket is also not conclusive. Absence of proof is not proof of absence. In the Maeda study PI4P, an accepted ligand, was also not detected. In the Prinz Schultz 2009 study, Fig S1 Osh4p did not sediment cholesterol containing liposomes efficiently either. In the Chung study only truncated ORP8 was used and full-length protein was not examined. Truncation was sufficient to abrogate photo-crosslinking of sterols to ORP8 in the Suchanek 2007 study. In any case, if anything ORP5 rather than ORP8 may be the homolog that would be more likely to display this activity. So, while these data argue against a sterol transfer role, we do not believe they are sufficient to completely discount the idea, particularly for ORP5, and all we are saying is that the question has not yet been resolved.
Overall comment: the authors must clearly define throughout the text what they mean by "lipid binding". The ORD surface can bind to lipids present in a membrane to associate (not specifically) with this membrane or can specifically extract and transfer a lipid (PS or PI4P) - This distinction is not often made in the manuscript or in the field in general. Yet this is quite fundamental..
We absolutely agree this is a fundamental distinction. For us lipid binding means an interaction with lipid that is sufficiently strong that it can mediate membrane recruitment for example. Binding can occur on the protein surface or in a pocket. Extraction and transfer require binding as a prerequisite, but go a step further, and is an activity attributed to pockets of lipid transfer domains and not the protein surface or lipid binding domains. We have now emphasized this distinction in the introduction, Pg 2-3 lines 95-104
Second, some structural data completely contradict some old or more recent claims and this should be said
This is now mentioned in the introduction, Pg 2 lines 85-94.
About the conclusion section, authors might consider two additional points.
- PI4P is a ligand for ORP5/ORP8 but also for OSBP and ORP1. Thus a lack of ORP5/8 might somehow impact PI4P level in the endosomal/lysosomal compartment. In turn this might change the activity of OSBP and/or ORP1 and sterol level in the limiting membrane of endosomes/lysosomes – recent papers showed intriguing links between OSBP and retromer or mTORC1 at endosome/lysosome levels . Authors should discuss this point.
We absolutely agree, and thank the reviewer for this point. We have now added to the discussion that changes in PI4P could impact the sterol transfer activity of other ORPs such as OSBP and ORP1L , Pg 17 lines 581-583.
- There is a special link between PS and sterol. These lipids tend to coalesce together (Fairn’s papers). Authors should shortly discuss whether a misdelivery of PS could in turn change how sterol is distributed in some compartments, again to interpret old data.
We thank the reviewer for this intriguing suggestion, and have added this point to Pg 17 lines 583-588. However, we do note that in cells that have a reduced PS synthase activity which results in 80% PS decrease, total cholesterol levels are unchanged and filipin (detect outer and inner leaflet cholesterol) staining looks normal. So changes in PS can influence the distribution of cholesterol and contribute to the phenotype but may not by itself explain the cholesterol accumulation in ORP5-depleted cells.
Last, authors should more precisely say what could be the sensor role of ORP5/8 – Because there is no mechanistic data on that, this concept remains quite obscure.
To us the use of the term sensing means that binding (but not extraction or transfer) of (oxy)sterols would alter the activity of the ORP5/8. A speculative mechanistic explanation would be something along the lines of (oxy)sterols binding to the outer surface of the ORP5/8 in a manner that induces a conformational change that either stimulates or inhibits it phospholipid transfer activity. If sterols or oxysterols bind within the pocket, even if weakly compared to phosphoinositides, they could alternatively competitively compete with phospholipids for binding, although from the discussion above this seems less likely. Finally, sterol binding might either stimulate post-translational modifications or through conformational change alter the interaction with other protein binding partners (for example NUP62) with or without affecting phospholipid transfer activity. This is now briefly discussed on Pg 17 lines 573-577
P2 line 61-66
These sentences are a little fuzzy, notably on how sterol transfer by Osh4/OSBP relies on PIPs . Prinz and co-workers showed that Osh4 transfers more rapidly sterol in the presence of PIP2 simply because PIP2 provides negative charge to membrane, not because PIP2 is a counterligand. Authors should provide the entire story : (i) the identification of Osh4 as a protein able to extract sterol or PI4P and not simply as a protein that binds to a PIP-rich membrane (de Saint-Jean et al, 2011) and (ii) the work on OSBP. Of note the study on OSBP did not show that OSBP transfers sterol against its gradient by counterexchange with PI4P. This has been only formally demonstrated using Osh4 by Moser von Filseck et al in Nature Com, 2015.
We have added a discussion of Saint-Jean and Moser von Filsek papers as suggested Pg 2 lines 61-71. To be fair, in the OSBP (Mesmin 2013) study they do claim to have shown that OSBP transfers sterol against its gradient by counter exchange with PI4P by showing the lipid changes in the Golgi and manipulating lipids in the ER with overexpression, siRNA (of Sac1) and the use of various OSBP mutants, lipid probes and drugs. However, we have softened our statements on the Mesmin study.
P2 line 73-74 : The authors must precise whether they have aligned sequences or compare structures to get this conclusion.
We have aligned sequences, as mentioned in the Figure legend. This is now more clearly stated in Pg 2 line 81
P2 line 80. I think that this amphipathic helix cannot help ORD5 to target LDs simply because this AH is part of the ORP ORD5 core structure and is deeply embedded inside this structure according to any robust molecular model based on homology with Osh6/Osh7. I think that the JCB paper on LD and ORP5 is convincing but there is a big problem in term of structural analysis. I will be very cautious on this.
We have added a note of caution about these data in lines pg 2 lines 85-90
Figure 1 : panel C. There is definitively no coiled-coil region in Osh6/Osh7 according to the PDB structures of Osh6. I think the figure must be corrected and that the corresponding paper in EMBO J must be re-discussed according to the structural data that have been obtained later by Maeda and co-workers.
The coiled-coil regions are now deleted from Figure 1 is now corrected and references to it changed on Pg 8 lines 246-247
Figure1 : panel A – I would say ORD signature motif
This is now changed in Figure 1.
P5 line 186 : typo
Now corrected on Pg 7 in new line 220
P6 –line 212 : this paper must be re-analyzed according to the structural data on Osh6 (see previous comment)
We now state the fragment used was initially thought to be a coiled-coil domain but was later shown to be a part of the ORD, Pg 8 lines 246-247
P6 – line 236 “dihydroergosterol “ à “dehydroergosterol”
Now corrected on Pg 8 in new line 271
P6 – line 249 : typo ‘the whether”
Now corrected on Pg 9 in new line 284
P6 – line 255 : typo ‘in-vitro’
Now corrected on Pg 9 in new line 297
P6 – line255-256 and following sentences in page 7 : To me, assays showing the binding of Osh6/Osh7 to phospholipid spotted on nitrocellulose or even included in liposomes are not indicative of an ability to regulate these phospholipids. Only the work of Maeda and co-workers showed first an ability of Osh6/7 to regulate PS. Also, back to my overall comment, I would say “ phospholipid encapsulation by ORP5” , “the role of Osh6 in capturing..” instead of “binding”
We have changed the wording to say “capturing” rather than binding in new lines 297-300. We softened the wording to say that these early studies hinted at a connection between ORP5 and 8 and phospholipids. Line 299.
P7 – line 267 – the two back-to-back papers were published in Science
Now corrected on Pg 9 in new line 310
P7 –line 300-304. It must be said that, comparatively to the DeCamilli’s paper, that this paper reported clear measurement of PS transfer from the ER to the PM and the dependence of this process on a PI4P gradient.
This is now highlighted in Pg 10 in new line 353-354
P7 – line 307 – I don’t understand the use of the word “co-exchanger”
This has now been changed to say that other PIPs might be exchanged for PS. Pg 10 line 361
P8 – line 318 : typo “asses”
Now corrected on P10 new line 373 (Nina)
P8 – line 345-360 – No doubt that the T Balla’s work is beautiful and decisive but please note that the De Camilli’s group had already replaced the PH domain of ORP5/8 by a FKBP module in its Science paper, showing that the recruitment of these proteins upon adding rapamycin elicits a change in PM PS and PI4P level, thereby validating the PS/PI4P exchange model. Authors should correct this paragraph.
We apologize for the oversight and thank the reviewer for catching this mistake. The two sections have been corrected in Pg 10. lines 340.346 and Pg 11 lines 404-410.
P9 –line 367 – typo “PIP5kIb”
Now corrected on Pg 11 line 426
P9 – line 380 – I think that ORP8 does not replace ORP5 but is recruited at the MCSs to work in addition to ORP5 when the level of PIP2 rises at the PM.
“Replaces” is changed to “joins” on Pg 12 in new line 439
Figure 2 : panel A : I would not draw a PH domain like that , binding two PIPs at the same time – I think this is wrong from a structural point of view and misleading.
Now corrected in new Figure 2
Please note that the names used to designate PIPs are not the same throughout the manuscript.
Now corrected throughout the text
Reviewer 2 Report
The review by Nina Criado Santos et al. is a very balanced and thorough review of the roles of two related lipid transfer proteins in normal cellular function and disease. These proteins, ORP5 and ORP8, have important roles in maintaining lipid homeostasis in cells, and their misregulation results in diseases including atherosclerosis and cancer. The authors have done an absolutely excellent job of presenting all of the published data on these important proteins, including contradictory results, while at the same time indicating how all of these disparate results could come together. I have only a few minor comments, detailed below.
Detailed comments
- Is it possible that the effect of ORP5 and ORP8 on cholesterol transport could be a result of their PI4P countertransfer function? In other words, could the perturbations in cholesterol levels be due to their efficient extraction of PI4P from the acceptor membrane, thus blocking the function of other cholesterol transport proteins that use a cholesterol-PI4P counterexchange mechanism between the ER and the same acceptor membrane?
- Lines 65-66. The authors state that “This counter-exchange mechanism proved later to serve as a model for other ORPs, including ORP5 and 8.” The authors should rather say that this counter-exchange mechanism was demonstrated for other ORPs, including ORP5, 8 and Osh6.
- Line 81. Should be “membranes”, plural.
- Line 186. Should change “However, exact the mechanisms….” to “However, the exact mechanisms…”
- Line 267. Should be “Science”, not “Nature”.
- Lines 357-360. The authors state: “Interestingly, neither PM PI(4,5)P2 nor cholesterol levels were changed, suggesting firstly that the effects of ORP5 on PM PI(4,5)P2 observed previously may be indirect [23], and secondly, that cholesterol is likely not transported by ORP5 or 8, at least not at the PM.” This sentence and the following one (lines 360-361) imply that there is a connection between cholesterol and PI(4,5)P2 transport by ORP5 and ORP8. If this is the case, the authors should make clear in what way they are coupled. If the authors are just grouping two lipids not directly affected by ORP5 and ORP8, it might be better to rewrite these sentences to treat cholesterol and PI(4,5)P2 separately.
- Line 516. It would be best to add “localization” to this phrase: “…requires PM PS for its localization and activity.” KRas has a C-terminal polybasic region that binds tightly to acid phospholipids, including PS, and since membrane binding is essential for its function, PS is therefore required for KRas activity as well, as stated in the reference given.
Author Response
Please find below our point-by-point response in italics. The page and line numbers correspond to the file with "tracked changes" on with the option "Show all revisions in line"
Reviewer 2
The review by Nina Criado Santos et al. is a very balanced and thorough review of the roles of two related lipid transfer proteins in normal cellular function and disease. These proteins, ORP5 and ORP8, have important roles in maintaining lipid homeostasis in cells, and their misregulation results in diseases including atherosclerosis and cancer. The authors have done an absolutely excellent job of presenting all of the published data on these important proteins, including contradictory results, while at the same time indicating how all of these disparate results could come together. I have only a few minor comments, detailed below.
We thank the reviewer for these positive comments.
Detailed comments
- Is it possible that the effect of ORP5 and ORP8 on cholesterol transport could be a result of their PI4P countertransfer function? In other words, could the perturbations in cholesterol levels be due to their efficient extraction of PI4P from the acceptor membrane, thus blocking the function of other cholesterol transport proteins that use a cholesterol-PI4P counterexchange mechanism between the ER and the same acceptor membrane?
Yes, absolutely. This is what we meant by indirect effects. Reviewer 1 also raised this point. We have now added that changes in PI4P might affect recruitment of OSBP and ORP1L and that this instead could indirectly drive sterol accumulation Pg 17 lines 581-583.
- Lines 65-66. The authors state that “This counter-exchange mechanism proved later to serve as a model for other ORPs, including ORP5 and 8.” The authors should rather say that this counter-exchange mechanism was demonstrated for other ORPs, including ORP5, 8 and Osh6.
This is now corrected on Pg.2 in new line 71- 72
- Line 81. Should be “membranes”, plural.
Now corrected in line 86 Pg.2
- Line 186. Should change “However, exact the mechanisms….” to “However, the exact mechanisms…”
Now corrected on Pg 7 in new line 220
- Line 267. Should be “Science”, not “Nature”.
Now corrected on Pg 9 in new line 310
- Lines 357-360. The authors state: “Interestingly, neither PM PI(4,5)P2 nor cholesterol levels were changed, suggesting firstly that the effects of ORP5 on PM PI(4,5)P2 observed previously may be indirect [23], and secondly, that cholesterol is likely not transported by ORP5 or 8, at least not at the PM.” This sentence and the following one (lines 360-361) imply that there is a connection between cholesterol and PI(4,5)P2 transport by ORP5 and ORP8. If this is the case, the authors should make clear in what way they are coupled. If the authors are just grouping two lipids not directly affected by ORP5 and ORP8, it might be better to rewrite these sentences to treat cholesterol and PI(4,5)P2 separately.
We now treat the two lipids separately, corrections on Pg 11 in new lines 415-419
- Line 516. It would be best to add “localization” to this phrase: “…requires PM PS for its localization and activity.” KRas has a C-terminal polybasic region that binds tightly to acid phospholipids, including PS, and since membrane binding is essential for its function, PS is therefore required for KRas activity as well, as stated in the reference given.
Now corrected on Pg 17 in new line 593
Reviewer 3 Report
The review by Santos et al. is very thorough in describing the role of ORP5 and ORP8 at various membrane contact sites. The review walks the readers through the first studies that initially suggested that ORP5 and ORP8 were involved in sterol sensing to more recent papers describing the role of ORP5 and ORT8 as lipid transport proteins. The review is very comprehensive in describing what is currently known in the field and as such it will be of great benefit to scientists studying lipid sensing, lipid transport, and the role that MCS have in regulating various processes in the cell.
I only have a few of minor comments:
- The first paragraph of the Introduction describes the importance of lipids and membrane composition in different cellular processes. It might also be useful to briefly mention the importance of lipid composition in the replication of viruses, positive-strand RNA viruses in particular.
- It would be great if the authors could point out the location of the amphipathic helix on the diagrams for ORP5 and ORP8 in Figure 1 as the helix is important to allow the proteins to simultaneously bind to two membranes.
- There are a few grammatical mistakes in the paper that should be fixed before publication.
Author Response
Please find below our point-by-point response in italics. The page and line numbers correspond to the file with "tracked changes" on with the option "Show all revisions in line"
Reviewer 3
The review by Santos et al. is very thorough in describing the role of ORP5 and ORP8 at various membrane contact sites. The review walks the readers through the first studies that initially suggested that ORP5 and ORP8 were involved in sterol sensing to more recent papers describing the role of ORP5 and ORT8 as lipid transport proteins. The review is very comprehensive in describing what is currently known in the field and as such it will be of great benefit to scientists studying lipid sensing, lipid transport, and the role that MCS have in regulating various processes in the cell.
We thank the reviewer for these positive comments
I only have a few of minor comments:
- The first paragraph of the Introduction describes the importance of lipids and membrane composition in different cellular processes. It might also be useful to briefly mention the importance of lipid composition in the replication of viruses, positive-strand RNA viruses in particular.
The importance for lipids in viral replication is now mentioned in Pg.1 line 31
- It would be great if the authors could point out the location of the amphipathic helix on the diagrams for ORP5 and ORP8 in Figure 1 as the helix is important to allow the proteins to simultaneously bind to two membranes.
Figure 1 now shows the location of putative amphipathic helices. Please also see discussion made by reviewer 1.
- There are a few grammatical mistakes in the paper that should be fixed before publication.
We apologize and have corrected many mistakes in the final draft (see also reviewer 1 and 2 comments)